# Skull base osteomyelitis: HBO as a therapeutic concept Effects on clinical and radiological results

**DOI:** 10.1007/s00405-024-09081-2

**Published:** 2024-11-29

**Authors:** Miriam Simon, Sven Dreyer, Insa Joost, Christian Rubbert, Jörg Schipper, Julia Kristin

**Affiliations:** 1 Department of Otorhinolaryngology, Duesseldorf University Hospital, Moorenstrasse 5, 40225 Duesseldorf, Germany; 2 Department of Orthopedics and Trauma Surgery, Duesseldorf University Hospital, Moorenstrasse 5, 40225 Duesseldorf, Germany; 3 Department of Medical Microbiology and Hospital Hygiene, Duesseldorf University Hospital, Moorenstrasse 5, 40225 Duesseldorf, Germany; 4 Department of Diagnostic and Interventional Radiology (Neuroradiology), Duesseldorf University Hospital, Moorenstrasse 5, 40225 Duesseldorf, Germany

**Keywords:** Skull base osteomyelitis, Hyperbaric oxygen therapy, Skull base surgery, Antibiotics, Clinical and morphological outcomes

## Abstract

**Introduction:**

Skull base osteomyelitis is a rare but potentially life-threatening disease. It usually occurs as a complication of severe otitis externa or infection in the nasopharynx, often in immunocompromised patients. The therapeutic strategy is complex, patient-specific and requires interdisciplinary cooperation.

**Material and method:**

A retrospective evaluation of all patients with skull base osteomyelitis at the Department of Otorhinolaryngology of the University Hospital Duesseldorf from 2006 to 2023 was carried out. It was investigated which factors, in addition to treatment regimens (antibiotic therapy with i.v./oral antibiotics, surgical debridement and HBO therapy) have an influence on the clinical, laboratory and morphological outcome of the patients.

**Results:**

A total of 42 patients who received interdisciplinary treatment in our clinic were included in the study, of whom 71.4% were male and 28.6% female. The tissue samples showed an inflammatory process, with detection of *Pseudomonas aeruginosa* in 68.4% of patients. A total of 61.9% of patients presented cranial nerve deficits, most frequently facial nerve palsy. A total of 66.7% of patients received HBO therapy. Of these, *n* = 20/23 patients (87%) with HBO improved and achieved regression in the follow-up imaging.

**Summary:**

Known patient-specific factors were confirmed and HBO was emphasized as an important component of the multimodal therapy concept. HBO appears to be justified and should continue to be included in the treatment regimen in the future. For this reason, patients with SBO should be sent to a center that offers HBO therapy.

## Introduction

Skull base osteomyelitis (SBO) is a rare but potentially life-threatening disease. There is no standardized classification to date, although the current literature most frequently describes a division into typical and atypical [1–4]. A typical SBO (TSBO) is more common and characteristically occurs in older patients with diabetes mellitus in terms of a dissemination of a malignant otitis externa caused by *Pseudomonas aeruginosa*. An atypical SBO (ASBO), also known as a central SBO, affects mainly the base of the sphenoid and occipital bones. It is usually not preceded by otitis [5].

In TSBO, the disease may initially begin as localized malignant otitis externa. As the infection progresses deeper, microorganisms enter the cartilage and bone and can then spread through natural gaps in the cartilage framework of the external auditory canal (fissures of Santorini) [1]. Spread to the temporomandibular joint is possible via the Huschke foramen. The phenomenon ultimately results in local bone destruction with localized marrow infiltration. The most frequent spread is then anteromedially to the infratemporal soft tissues, the tip of the petrous bone and the clivus [1]. Progression usually occurs with an inconsistent treatment regime and can manifest itself as pronounced otorrhea, increasing pain or even cranial nerve deficits [6]. The 7th cranial nerve (facial nerve) is particularly affected in this case due to anatomical conditions. According to prior case series, between 7% and 32% of patients with SBO develop facial nerve palsy [6–11]. In most cases, additional clinical deficits of other cranial nerves in the caudal group (IX-XII) indicates disease progression [6].

ASBO is an infection of the ethmoid, sphenoid, occipital, or temporal bones that form the skull base. The infection can spread along the soft tissues of the skull base. Infiltration of the Haverisan canal allows it to spread along the cancellous bone. ASBO is most frequently the result of an advanced paranasal sinus infection with corresponding spread. More rarely, the cause is iatrogenic in cases following endoscopic nose surgery or paranasal sinus surgery. In some cases, the cause cannot be definitively determined [12]. Because of the lack of otogenic causes, some authors call ASBO sinonasal or nonotogenic SBO. The clinical features are often less specific: headaches, cranial neuropathies and sinunasal symptoms [2, 13].

However, the distinction between TSBO and ASBO is not always completely clear. TSBO and ASBO can both involve the clivus. In this case, only a clinical differentiation can be made, especially otoscopically. Symptoms and signs of inflammation of the external auditory canal or middle ear usually indicate the presence of a typical SBO [12]. Additionally, patients may have suffered an occult or sometimes incompletely treated TSBO prior to diagnosis. Inflammation may spread from lateral to medial or vice versa [14].

The lack of a guideline-recommended standardized treatment is probably due to the small number of cases of this disease. Consequently, therapeutic strategies are complex, patient-specific, and require interdisciplinary collaboration. The current treatment regime is based on three pillars: antibiotics, surgical intervention, and if available hyperbaric oxygen therapy (HBO).

The aim of this study was to evaluate the influence and meaning of hyperbaric oxygen therapy on therapeutic outcomes. Decisive features for assessing the outcome of patients are the changes or improvements in clinical symptoms, particularly cranial nerve deficits. Another factor that was compared and evaluated was the imaging at the beginning and after completion of hyperbaric oxygen therapy.

## Materials and methods

A retrospective analysis of all patients treated with SBO (TSBO and ASBO) at the Department of Otorhinolaryngology at our hospital from 2006 to 2023 was performed. The analysis included 42 patients aged ≥ 18 years, who were divided into two groups: typical and atypical skull base osteomyelitis. The effects of hyperbaric oxygen therapy as a treatment option on the clinical and morphological outcomes of patients were observed.

Local Ethics Committee approval was obtained (No: 2024–2939).

### HBO

The number of HBO sessions for each patient was evaluated.

**Fig. 1:**
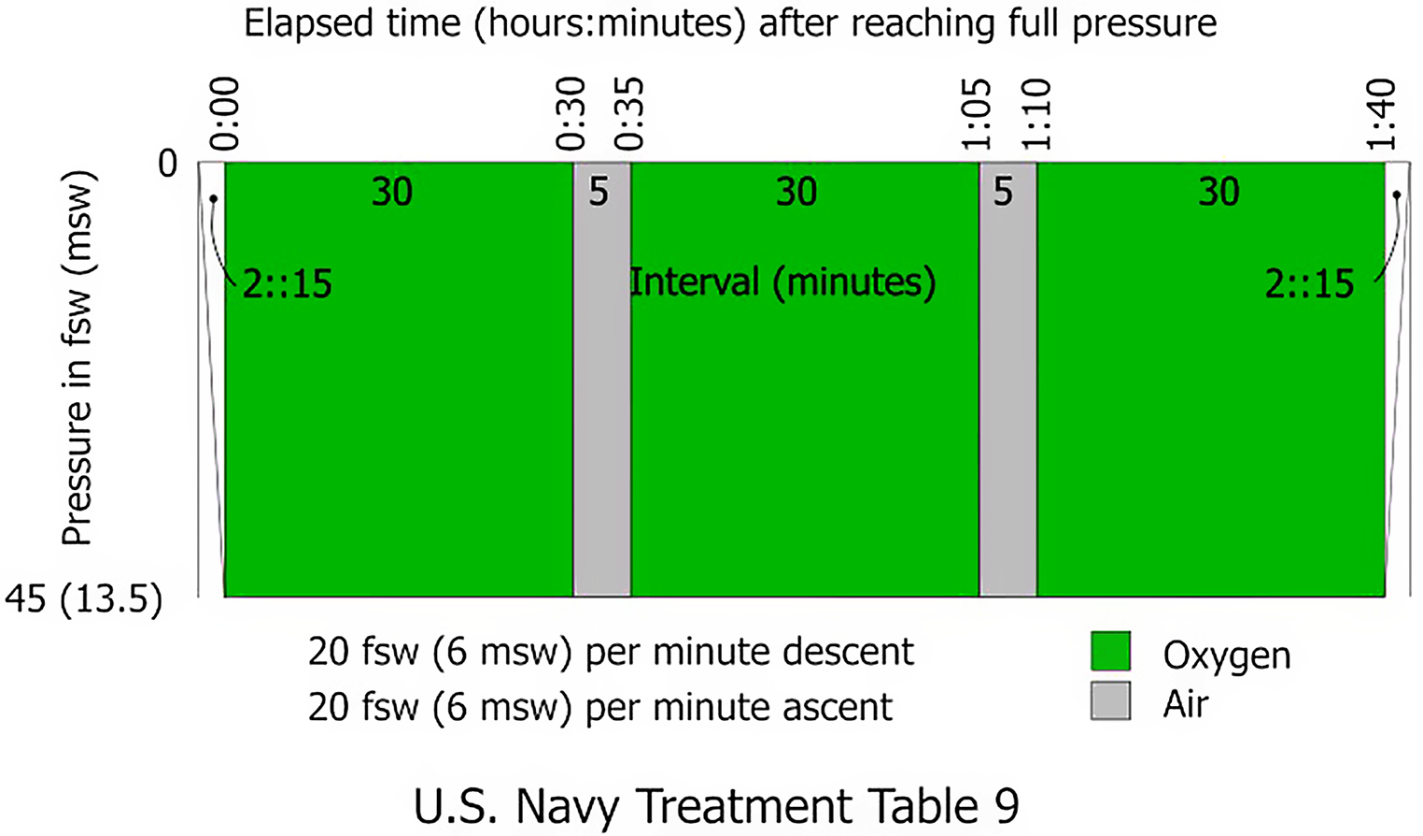
Schematic representation of the process in the pressure chamber [30]

The course of HBO therapy is explained using the example of a session with 25 units (Fig. 1). The units of HBO were administered using therapy scheme (TS) 240 − 90 according to the GTÜM e. V. guidelines. The 25 HBO were administered over 25 days according to the predesigned therapy scheme (Monday to Friday, 5 − 2 rhythm).

One HBO therapy unit TS240-90 comprised a compression phase breathing air for 11 min up to 240 kPa absolute pressure (corresponding to a water depth of 14 m). This exposure was followed by an isopression phase of 110 min at 2.4 bar pressure. During this phase, 100% oxygen was administered for 3 × 30 min. Between each of the 3 phases of breathing 100% O2 were 2 5–10-minute phases of air breathing (“O2 break”). Each HBO unit ended with a decompression phase of 10 min of breathing 100% O2. The total duration of therapy was 131 min, and the total time spent breathing 100% O2 was 100 min, of which 90 min were at a constant positive pressure of 2.4 bar absolute.

### Clinical symptoms

The descriptive data, the clinical symptoms of *n* = 42 patients including cranial nerve deficits, and the results of the microbiological analysis were recorded and analyzed.

### MR and CT imaging

The MR images of *n* = 30 patients in total (with/without HBO) were considered, as both the initial image and the image after therapy were available. The images were assessed by the local neuroradiology department according to the extent of the skull base osteomyelitis.

In our clinic, MRI controls are usually performed 8–12 weeks after the end of HBO, followed by a repeat imaging session after 6–12 months (depending on the clinical symptoms). The images are then discussed at the weekly interdisciplinary radiology board with a statement from colleagues in neuroradiology. The MR imaging is performed with contrast medium (3D T1-weighted MPRAGE (TR = 2.0s, TE = 2.45ms, TI = 900ms, flip angle = 8°, field of view = 250 × 250 mm, slices = 160, voxel size = 1 mm³).

The focus of the imaging evaluation is on the extent of the inflammation or the intensity of the contrast enhancement or resclerosis of bony defects in computed tomography. A computer tomography can be added during the procedure.

### Statistics

Microsoft Excel Version 16.81 and “IBM SPSS Statistics Version 29” were used for the statistical analysis of the dataset.

## Results

A total of 42 patients with SBO who received interdisciplinary treatment in our clinic were included in our study.

There has been an increase in the number of patients with SBO at our university hospital, over the time, particularly in the last 5 years. Nine patients were identified in the period from 2014 to 2018, whereas 23 patients were discovered to have SBO from 2019 to 2023. Of the 42 patients assessed, 36 had TSBO and 6 had ASBO. Patients with SBO had an average age of 70.2 years (SD ± 11.3). Patients with TSBO were slightly younger (69.8 years of age (SD ± 11.8)) and patients with ASBO were slightly older (74.3 years of age (SD ± 6.7)). Regarding the distribution by sex, SBO generally occurred in 71.4% of males and 28.6% of females. Among those affected, more women than men had a TSBO (91.6% females vs. 83.3% males).

In the group of patients with TSBO, 86.1% of the primary symptoms were ear symptoms as follows: otorrhea, otalgia and hearing loss. In contrast, only 13.9% of patients experienced diffuse headaches. The distribution in the ASBO group was less clear. Despite being assigned to an ASBO group, 66.7% of patients had ear symptoms and 33.3% of patients had diffuse headaches.

Approximately 60% of all patients presented cranial nerve palsies. A distinction was made between facial nerve palsy and palsy of the caudal cranial nerve group (IX-XII). In the paresis group, facial nerve palsy was found in 73.1% (*n* = 19/26) of the patients. A loss of one or more nerves in the caudal group was found in 42.3% (*n* = 11/26). Regarding known risk factors, 66.7% (*n* = 28/42) of patients had diabetes as an additional disease (types 1 and 2).

Surgery was performed on all included patients. Some patients were only sampled in the nasopharyngeal region, whereas others underwent more extensive surgeries, such as petrosectomy, in terms of a necrosectomy. The decision regarding the extent of the surgery was made by considering the MRI / CT scan and clinical symptoms. The material obtained from the surgery was examined both microbiologically and pathologically. In the microbiological examination, 68.4% (*n* = 26/38) of the patients were a positive for P. aeruginosa. No documentation of the pathogen was found for 4 patients. All included patients received pathogen- and resistogram-specific intravenous antibiotic therapy over a period of at least 6 weeks.

HBO therapy was carried out in 24 patients. The exact documentation of the HBO therapy was insufficient for 6 patients, so they had to be excluded from the evaluation. Thus, the total number to be considered had to be reduced from *n* = 42 to *n* = 36. As a result, 66.7% (*n* = 24/36) of the patients received HBO therapy, and 33.3% (*n* = 12/36) did not. HBO therapy was not carried out in patients with pronounced comorbidity and an overly advanced clinical picture, who were further treated in the palliative setting. This decision was always made considering all the findings, in consultation with the specialist disciplines that treated the respective comorbidities and, of course, with the patients and their family members. Another reason for the lack of therapy initiation was simply refusal by the patient. The mean value of the HBO sessions performed was 20.2 (SD ± 8.4). The highest number of sessions was 35, and the lowest was 2.

**Fig. 2:**
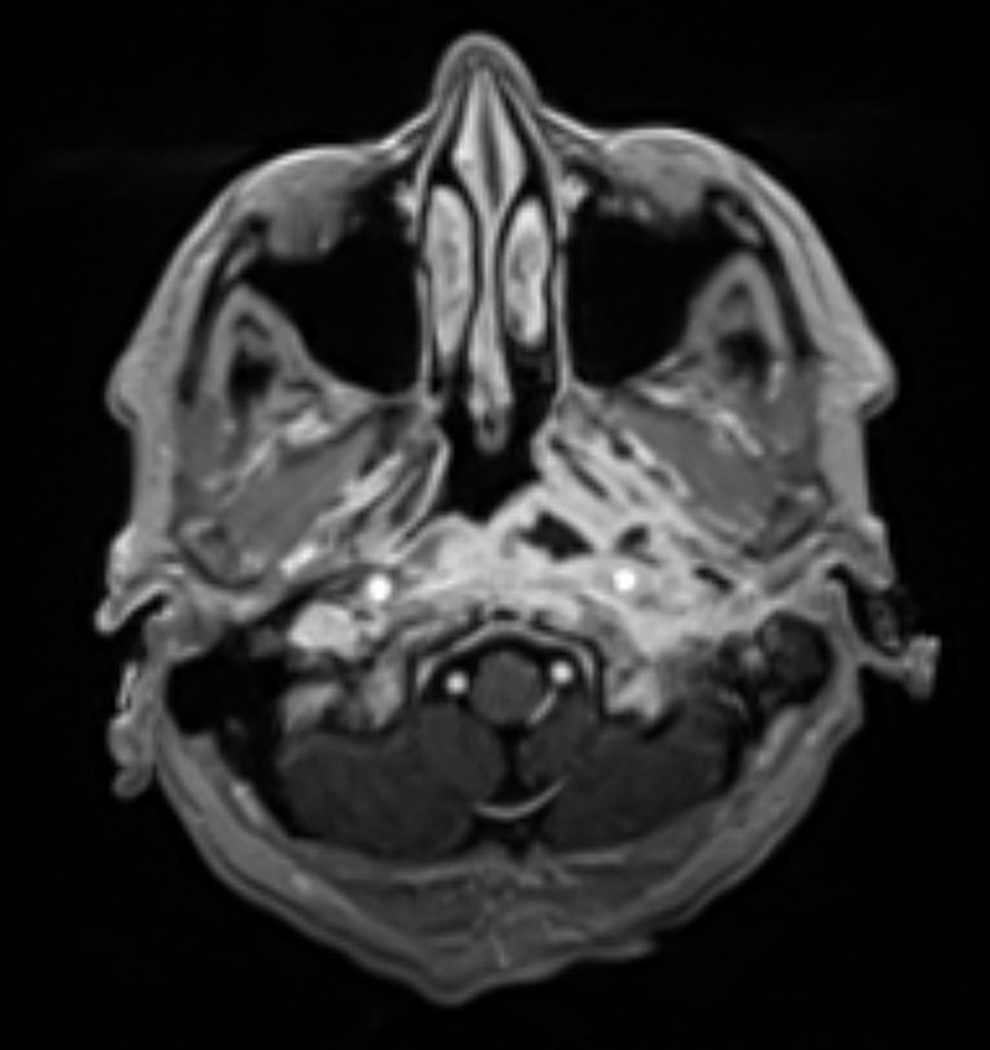
MRI examination at the start of treatment (TSBO)

**Fig. 3:**
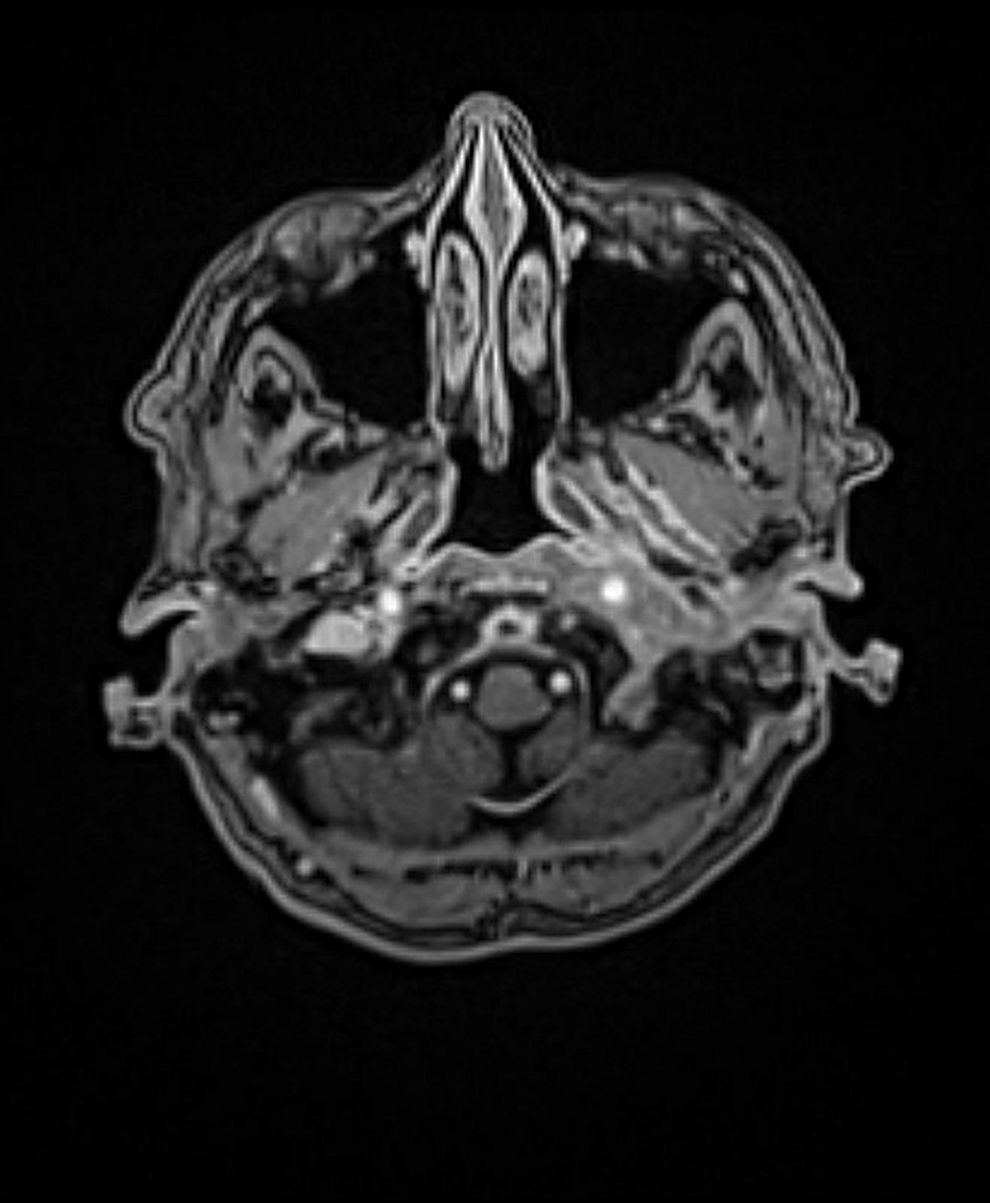
MRI examination after 6 months (TSBO)

**Fig. 4:**
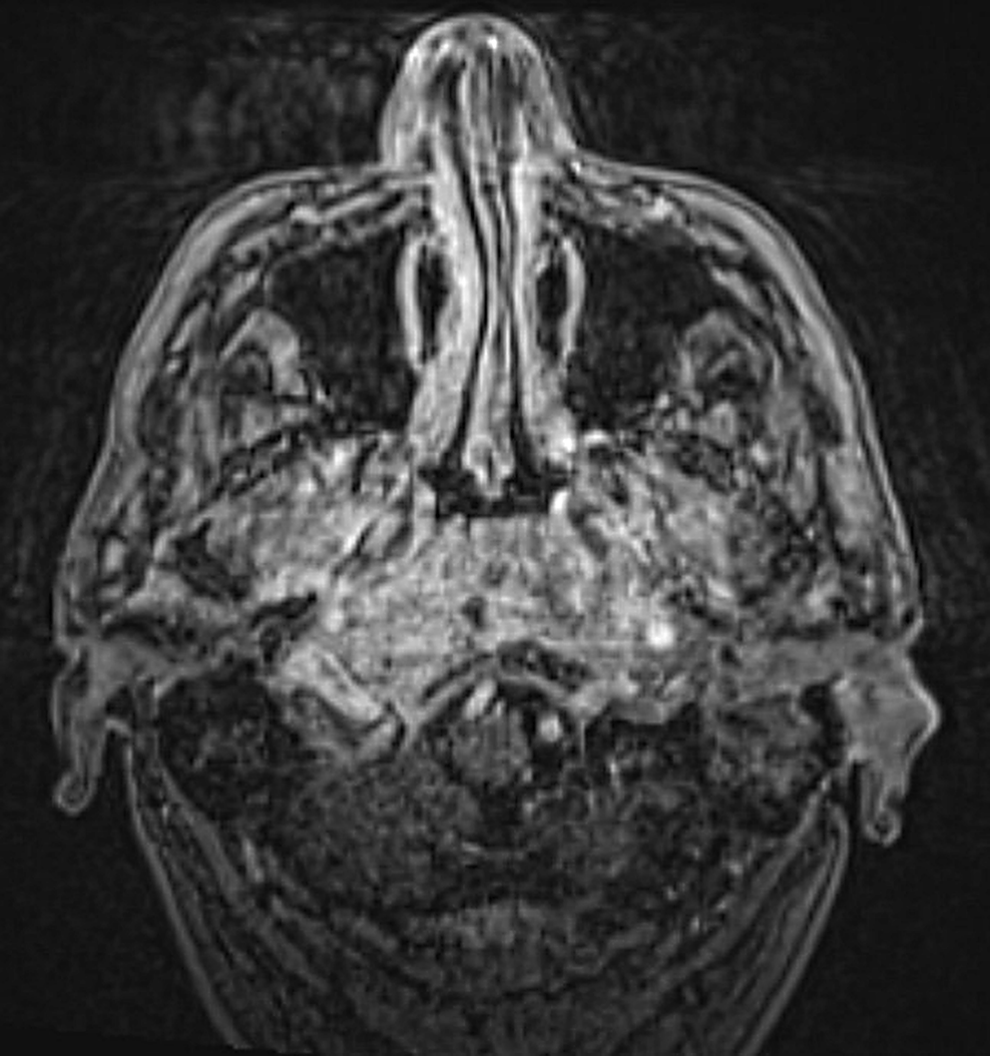
MRI examination at the start of treatment (ASBO)

**Fig. 5:**
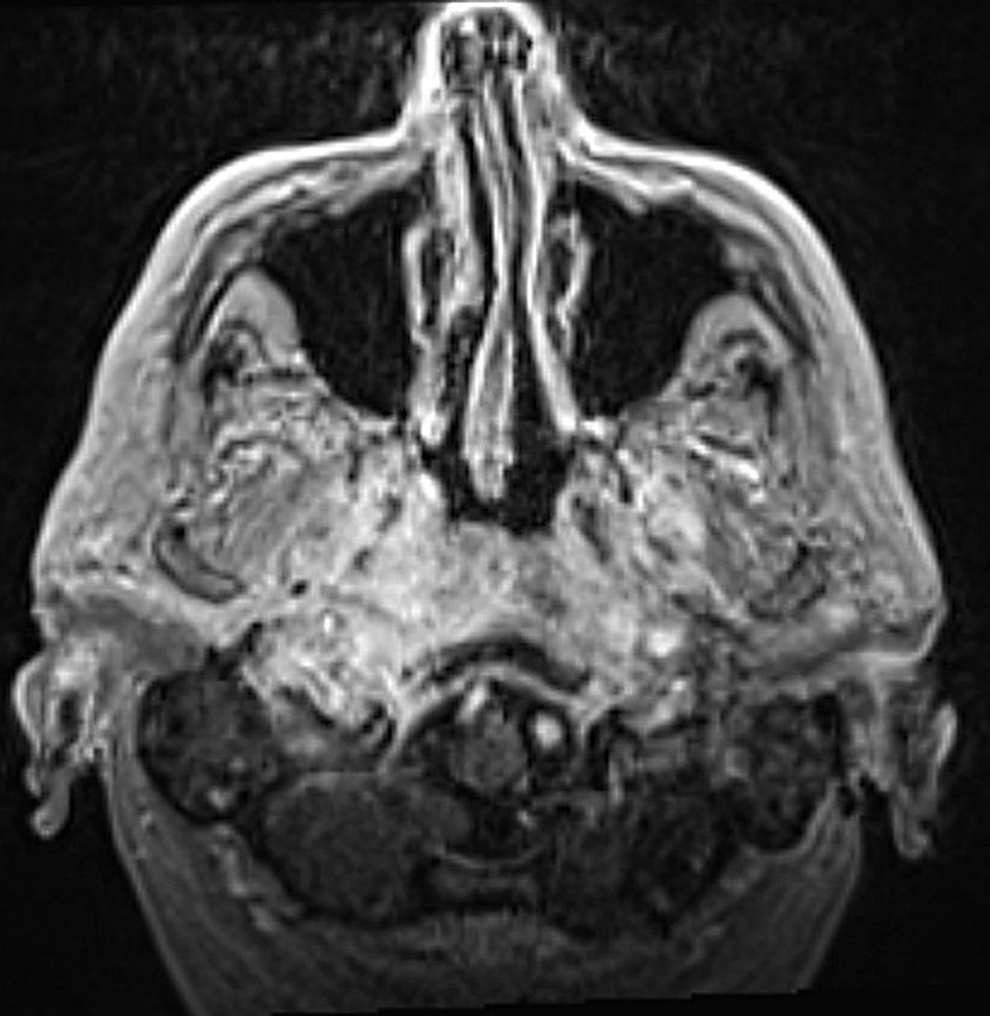
MRI examination after 6 months (ASBO)

The course of the disease was assessed based on the patient’s clinical symptoms and the imaging results. At the start of treatment, typical SBO often manifests as an extension of a known external otitis. This process can extend into the nasopharynx and is characterized by swelling and contrast agent uptake (Fig. 2). During therapy, a decreasing accumulation of contrast enhancement and less tissue swelling is expected due to the inflammatory process (Fig. 3). As previously mentioned, atypical skull base osteomyelitis is also described as a central spread of inflammation. The initial image often shows a mass effect and solid accumulation of contrast enhancement in and around the clivus and an infiltration with possible extension into the nasopharynx (Fig. 4). Over time, a decrease in the contrast enhancement and a decrease in other inflammatory formations, such as abscesses, can also be observed (Fig. 5).

With respect to the morphologic course of the disease, all patients with an MRI control as the starting and end point were be considered. Among the 24 HBO-treated-patients, 87% achieved regression to cure, 8.7% experienced progression, and 4.3% died. Among the 7 patients without HBO (existing contraindication, death, refusal), 57.1% experienced regression to cure, 28.6% experienced progression, and 14.3% died. Both patient deaths were due to a spreading inflammatory process that resulted in sepsis caused by the SBO. However, deaths due to many comorbidities, e.g., cardiological diseases, have also been reported. In patients with and without HBO, there was no statistically significant difference in the number of MRI scans of regression to cure and progression (U = 32.000, Z=-1.477, *p* =.140). Similarly, there was no significant difference between HBO and no HBO in terms of regression to cure (progression) and mortality (U = 16.000, Z=-1.127, *p* =.260; U = 4.000, Z = 0.000, *p* = 1.000).

## Discussion

As previously mentioned, there is currently no generally applicable treatment for the increasing number of patients with SBO. Therefore, sufficient attention should be given to this potentially life-threatening clinical disease, and patients should not be denied a presumably helpful therapy.

However, why have so many patients been diagnosed with SBO in our clinic and why has the number of cases increased in the last 5 years in particular? The clinical picture is becoming increasingly present, also evidenced by the repeatedly changing nomenclature over the years. Pervical Pott was the first individual to describe osteomyelitis of the cranial bones in 1775 [15]. This condition was describe in a patient with a subpericranial abscess after frontal bone injury. Over time, it became increasingly clear that such an infection was not the result of trauma but rather the spread of infection to neighboring structures, such as the paranasal sinuses. In 1959, Meltzer and Kelemen first described SBO in patients with burn injuries and osteomyelitis of the external auditory canal [16]. SBO is not only the cause of an inflammatory process of the external auditory canal, but it is also a consequence of inflammation of the face, nose, paranasal sinuses, oral cavity and pharynx [17, 20, 21]. 

A key aspect for this increase could be the demographic changes with increasing patient age and a corresponding increase in comorbidities, such as diabetes [17]. According to the latest estimates, the incidence and prevalence of diabetes (type 1 or type 2) are 1.2% for all age groups and 7.2% for the 18-79-year-old age group [18]. The prevalence of type 2 diabetes is expected to have risen by 54–77% by 2040 (in people aged 18 years and over) [19]. In the current cohort, the average age was 70.2 years. Considering the classification into typical vs. atypical skull base osteomyelitis, these figures are consistent with the assumption that TSBO occurs preferentially in an older cohort [13]. 

A comparison of TSBO and ASBO revealed that the age of the youngest included patient with an ASBO was 65 years, which was significantly older than the age of the youngest patient with TSBO (49 years). This difference can be explained, on the one hand, by the classification in the literature with the rather conjectural distribution of patient age, on the other hand, by the size of the cohort with ASBO (*n* = 6/42) which is too small to draw generally valid generalizable conclusion.

Germany has 22 pressure chambers, which are divided into 3 different categories (1: with an emergency center, 2: with 24 h of on-call service, and 3: with limited service). As a location within a radius of 70–80 km to the next pressure chamber (category 1) for hyperbaric oxygen therapy, we are the only maximum care hospital that has both, a certified skull base center and a pressure chamber. This unique situation leads to an increasing number of referrals.

Focusing on hyperbaric oxygen therapy, the first question to ask is how HBO can help at all. HBO maximizes plasma-based oxygenation and leads to intermittent hyperoxia, which is necessary for collagen synthesis and angiogenesis. Leukocyte-mediated bacterial killing is enhanced, as is the effectiveness of various antibiotics, e.g., oxygen-dependent aminoglycoside transport through bacterial cell walls. HBO kills anaerobic bacteria directly and indirectly and promotes the oxygen-dependent osteoclastic resorption of necrotic bone. The reduction in edema, inflammation and compartment pressure is also significant, which ultimately leads to an improved blood circulation.

The number of treatments required depends on the severity of the disease [20]. Notably, our group was heterogeneous in terms of the implementation of the respective therapies. For example, some patients had undergone complete surgical treatment, and others had received prolonged antibiotic treatment. There were also different durations of HBO treatment. Antibiotic treatment according to the antibiogram is based on the chance of revascularization of the bone and should therefore be carried out for at least 4–6 weeks [21]. The respective supplementary regimens of HBO therapy have already been mentioned. The number of sessions is still inconsistent. In the case of diabetic foot disease, it seems simple: a reduction in the clinical picture (extent of necrosis, etc.) indicates a clear response to therapy and can make it possible to plan the end of therapy [22]. There are guidelines for many other diseases for which HBO therapy may be indicated. However, there are no such guidelines for SBO. As already mentioned, reducing the clinical manifestations of SBO is not trivial, and we are unable to obtain a clinical view of the skull base without imaging. Consequently, the minimum duration of systematic antibiotic treatment is currently being used as a guide [23]. This duration is usually approximately 30 sessions, with the assumption of at least 4 weeks of intravenous antibiotics. As previously mentioned, the mean number of the HBO sessions performed in our clinic was 20.2 (SD ± 8.4). The largest number of sessions was 35, and the smallest was 2. In 2023 and 2024, most patients had 30 sessions, which corresponded to the duration of i.v. antibiotics.

Other ENT-related illnesses with HBO as a potential treatment option include necrotizing fasciitis, sudden deafness or acute noise trauma. However, opinions on the success of therapy and the requirements for the latter two vary widely [24, 25]. 

The heterogeneity of the groups also explains the lack of significance regarding the outcome of the patients in the statistical analysis. Unfortunately, this feature reduces the comparability. A pure comparative study (patients who receive HBO and patients who do not receive HBO) is not easy to implement for ethical reasons because a possible therapy would be withheld, i.e., HBO, from the patient. This is particularly strict because skull base osteomyelitis is a disease with a known mortality rate. Therefore, only a comparison with the data available in the literature of patients who have not received HBO, e.g., due to a nonexistent pressure chamber or other reasons, as well as a comparison with patients who refuse HBO, is possible. Patients who have a contraindication for HBO are usually so severely ill that they represent a group that can be assessed only to a limited extent as a comparison. Compared with the current literature, the number of cases in this study was very high (*n* = 42) over the period from 2006 to 2023. Akhtar et al. reported a total case number of 15 patients over a longer observation period, and many papers are meta-analyses [2, 26]. Even if this finding is not confirmed based on statistical significance, a trend is visible. Among the patients who have received HBO therapy, regression to healing occurred in 87% of cases; by comparison, of those without HBO, regression to healing was achieved in 57.1% of cases. A similar trend was observed regarding mortality, with patient deaths in 4.3% vs. 14.3% of cases in the group with vs. without HBO therapy respectively. In another study, the healing rate with regard to signs of inflammation was 100% (*n* = 8/8), and the rate of complete functional restitution was 75% (*n* = 6/8) (two patients who refused any therapy were not included here) [27]. 

Since HBO therapy is an easily accessible therapy with few side effects if a pressure chamber is available, there are few absolute contraindications to its use (e.g., untreated tension pneumothorax, various ongoing chemotherapies), and there are usually no problems with the German health insurance covering the costs if this is explicitly justified in the inpatient case. This further therapy option should continue to be used if possible [28]. To date, HBO has only been a fixed part of the benefits catalog of statutory health insurance in Germany for emergency indications and diabetic foot syndrome and can therefore generally not be billed as an outpatient medical service at the expense of statutory health insurance. A different situation is found for patients in an inpatient setting. Here, the costs of the indications for diving accident, gas embolism, carbon monoxide poisoning, and neuroblastoma stage IV are covered by the health insurance funds as special charge additional to the flat rates per case (DRG) following a positive decision by the Federal Joint Committee (G-BA). All other indications, including the therapy concept for SBO, still require an individual application for reimbursement from the respective health insurance company. Reimbursement is generally guaranteed in cases where there is no alternative therapy, the disease has progressed under previous therapy, or the disease is life-threatening or severely restricts the patient’s quality of life [29]. 

Nevertheless, the results of this study suggest that patients with advanced SBO benefit from adjuvant HBO therapy. Additionally, mortality and morbidity appear to be improved with this intervention. In particular, the regression of existing cranial nerve palsies is positively influenced. In general, all treatment options should be exhausted for life-threatening diseases of this type, and patients should be transferred to appropriately equipped centers if necessary.

## Summary

In summary, known patient-specific factors were confirmed as risk factors and HBO was emphasized as an important component of the multimodal therapy concept. The expansion of interdisciplinary cooperation also appears to be essential in the future. HBO has its rightful place as a pillar of therapy and should continue to be part of the interdisciplinary three-part therapy concept in the future.

## Data Availability

No datasets were generated or analysed during the current study.
